# Resolutions Adopted by the National Dental Association at the Annual Session of 1898, and Other Miscellaneous Items

**Published:** 1898-11

**Authors:** 


### Resolutions Adopted by the National Dental Association at the Annual Session of 1898, and Other Miscellaneous Items.

Resolution adopted by Executive Committee and National Dental Association in regard to the proposed amendment to the patent law.

WHEREAS, The Supreme Court having already declared all such patents as are contemplated in this move to amend the patent law invalid, which makes all such amendments unnecessary, and

WHEREAS, The constant agitation of this question is detrimental to the best interests of organization in existance, be it

*Resolved*, That the National Dental Association, now in session, disapproves of any further work in this direction, and recommends that the whole question be dropped as unwise and unnecessary.

The following resolutions were offered by Dr. C. L. Goddard in relation to the appointment of Dental Surgeons to the Army and Navy:

*Resolved,* That this Association approves and endorses the movement for the appointment of Dentists in the Army.

*Resolved,* That a committee of five be appointed by the President to represent this Association in procuring from Congress the necessary legislation.

The resolutions were referred to the Executive Committee which, at a later session, reported as follows:

WHEREAS, The National Dental Association has appointed a committee to take charge of and have full control of the subject of legislation concerning the employment of Dental Surgeons in the Army and Navy,

*Resolved,* That this Association deprecates any independent action on the part of State and local societies or individuals without the approval of said committee. Adopted.

The committee appointed consists of Drs. Finley, Donnally and Butler.

In regard to the International Dental Congress, Paris, 1900, the following resolution was offered by Dr. A. W. Harlan and unanimously adopted:

WHEREAS, Provision has been made for the holding of an International Dental Congress in Paris, and committees have already been appointed to organize such a Congress, and that it is the will of said committees that the National Dental Association appoint a committee to co-operate with those committees in making the Congress a success; therefore, be it

*Resolved,* That a General Committee of fifteen be appointed by the President to co-operate with the General Committee in Paris, such committee to have power to add to its number, not to exceed twenty-five names in the United States; and that said committee, when organized, be empowered to adopt rules and regulations such as will insure the success of the Congress. The present President and Vice-Presidents of this body shall be members of this committee. After this committee has organized by the selection of a chairman and secretary its acts shall be final, and after the close of the Congress said committee shall present a report of its work to this Association.

At a subsequent session the committee of fifteen, together with the President and three Vice-Presidents, was appointed as follows: A. W. Harlan, Chicago; A. H. Fuller, St. Louis; H. J. McKellops, St. Louis; J. Taft, Cincinnati; H. A. Smith, Cincinnati; W. W. Walker, New York; Jas. McManus, Hartford; W. C. Barrett, Buffalo; T. W. Brophy, Chicago; B. Holly Smith, Baltimore; W. E. Griswold, Denver; C. L. Goddard, San Francisco; L. L. Dunbar, San Francisco; H. W. Morgan, Nashville; Frank Holland, Atlanta; E. C. Kirk, Philadelphia; J. D. Patterson, Kansas City, Mo.; Thomas Fillebrown, Boston, Mass.; Thomas E. Weeks, Minneapolis.

The Committee on the President's Address having recommended the adoption of a Code of Ethics, the chair appointed Drs. H. A. Smith, Frank Holland and H. W. Morgan a committee to formulate a Code of Ethics for this Association.

The Committee on National Museum and Library reported that having visited the institution in the interests of the Association, they had been cordially received, finding the officials interested in the work and expressing a willingness to do their utmost to build up the dental sections. A highly creditable amount of work in accumulating specimens has been accomplished, while the library contains the best and most complete collection of dental literature in the world. But few men in the profession appreciate this great work in elevating dentistry in the estimation of scientific men.

The recommendation of Surgeon-General George M. Sternburg endorsed by the Secretary of War, for the employment of a Dental Pathologist passed the Senate, but failed in committee of conference. The recommendation will, however, be renewed and submitted to Congress at its next session.

CHAIRMEN AND SECRETARIES OF SECTIONS.

Sec. I. I. N. Broomell, Chairman; Wm. E. Walker, Sec'y.
Sec. II. S. H. Guilford, Chairman; M. F. Finley, Sec'y.
Sec. III. J. Y. Crawford, Chairman; Frank Holland, Sec'y.
Sec. IV. T. L. James, Chairman; L. L. Dunbar, Sec'y.
Sec. V. J. S. Cassidy, Chairman; A. W. Harlan, Sec'y.
Sec. VI. J. D. Patterson, Chairman; L. E. Custer, Sec'y.

Sec. VII. W. C. Barrett, Chairman; W. F. Lewis, Sec'y.

Sec. VIII. J. Taft, Chairman; H. R. McFadden, Sec'y.

Sec. IX. V. H. Jackson, Chairman; C. L. Goddard, Sec'y.

Sec. X. H. J. McKellops, Chairman; M. B. Culver, Sec'y.

Dr. A. W. Harlan offered the following standing resolutions, which were unanimously adopted:

*Resolved*, The Secretaries of Sections are required to forward to the Chairman of the Executive Committee—sixty days before the annual meeting—the titles of papers to be submitted, with the names of the authors, in order that they may appear on the official program.

*Resolved*, The Executive Committee shall prepare and mail to the members an official program, at least twenty days before the annual meeting. This program shall have a list of the hotels, the place of meeting and the railway and steamboat facilities for getting to the place of meeting from the principal points in the United States. This notice shall be mailed to the journals at least one month before the annual meeting.

Dr. Molyneaux offered the following:

*Resolved*, That a committee of three be appointed who shall prepare amendments to our Constitution, providing for an Executive Council, to whom all miscellaneous business shall be submitted without discussion, before final action is taken.

This resolution was adopted.

Dr. Cassidy offered the following as a standing resolution, which was adopted:

*Resolved*, That the out-going President be a member of the Executive Council.

The Chair appointed as the other two members of the Executive Council for the coming year, Drs. Grant Molyneaux and John S. Marshall.

### RESULT OF THE ELECTIONS.

President, from the East, Dr. H. J. Burkhart, Batavia, N. Y.; Vice-President, from the East, Dr. S. H. Guilford, Philadelphia; Vice-President, from the West, Dr. Thomas E. Weeks, Minneapolis; Vice-President, from the South, Dr. B. Holly Smith, Baltimore.

(Age alone confers seniority among the Vice-Presidents).

The following officers were unanimously re-elected to their several positions: George H. Cushing, Recording Sec'y; Wm. Ernest Walker, Asst. Recording Sec'y; Emma Eames Chase, Corresponding Sec'y.

Drs. G. Henry, H. W. Morgan, S. Butler and J. Y. Crawford were elected members of the Executive Committee in the places of Drs. C. N. Peirce, W. P. Dickinson and George Eubank, whose terms expire with the present meeting.

Niagara Falls and Boston were placed in nomination for the place of meeting, Niagara Falls being chosen. By a unanimous vote the time of meeting was changed to the first Tuesday in August.

The Executive Committee appointed Dr. L. E. Custer, of Dayton, Ohio, to deliver a general address on "Electricity," and Drs. T. W. Brophy and Thomas Fillebrown to deliver addresses on "The Surgical Treatment of Cleft Palate."

Publication Committee, C. N. Johnson, Chicago; C. N. Peirce, Philadelphia.

Local Committee of Arrangements, C. A. Butler, D. F. Bentley, Buffalo, and M. O. Cooley.

Committee on National Museum, Williams Donnalley, Washington City; Henry W. Morgan, Nashville, and John S. Marshall, Chicago.

AMENDMENTS TO THE CONSTITUTION.

The proposed amendments to the Constitution, laid over from last year, (1) by Dr. J. Y. Crawford, to change the name of the Association to "The American Association of Dental Surgeons;" (2) by Dr. S. H. Guilford, to change the name to "The American Association of Dental Science," and (3) by Dr. Stellwagen to "The American Stomatological Association," were all tabled.

The following amendments, proposed by Dr. M. F. Finley, lay over for one session, and were then unanimously adopted:

Amend Sec. 1, Art. IV., by inserting after the third word in the first line, "and the names of the Territories enumerated in Sec. 3, Art. XII."

Amend Sec. 3, Art. III., by inserting after the first word of the third line, "and Territorial."

The following amendment, offered by Dr. H. J. Burkhart, lay over one session, and was then unanimously adopted:

Amend Article VI., second paragraph of Section 3, to read, "commencing with 1897 the President shall be chosen from the division in which the next meeting is to be held."

The following amendments, embodying the suggestions in the President's Address and recommended by the committee on the address, were read by Dr. H. W. Morgan, but were not acted upon:

Article VI. shall be amended so as to read, "the President shall be chosen from the division in which the next annual meeting is to be held."

Article III., Sec. 3, shall be changed so as to read as follows: "They shall be chosen in any manner that their Association may see proper."

In Article IV., Sec. 1, the word ten shall be changed so as to read six.

The following amendments, offered by Dr. Laurence Leonard, lie over for one year:

*Resolved*, That Art. V. be amended by adding after the word dollars, "the receipt for which will entitle the holder to all the privileges of the floor, including general meetings of the Executive Committee."

Art. VIII., Sec. 1, after the words, "Association to whom," add "and before whom all business matters shall come."

Art. VIII., create Sec. 10. The Executive Committee shall elect a president and secretary, and shall hold daily general sessions during the meeting of the Association.

Create Article XV. as follows: Section 1. After 1899 nothing shall come before the general sessions of the Association except the President's Address, Announcements, Election of Officers, selection of place of meeting, and the reading and discussion of scientific papers endorsed by the Sections.

Sec. 2. Anything in conflict with this article is hereby repealed.

A communication was received from the Northern Illinois Dental Society reporting the adoption by that body of resolutions looking toward a remedy for the existing evils regarding the Inter-State practice of dentistry, and requesting the National Dental Association to work in securing such modifications of the dental laws of the various States as shall enable competent practitioners to remove from one State to another without being compelled to submit to provisions which are eminently unfair to large numbers of capable dentists.

The committee was referred to Section II.

